# A comparative analysis of family-based and population-based association tests using whole genome sequence data

**DOI:** 10.1186/1753-6561-8-S1-S33

**Published:** 2014-06-17

**Authors:** Jin J Zhou, Wai-Ki Yip, Michael H Cho, Dandi Qiao, Merry-Lynn N McDonald, Nan M Laird

**Affiliations:** 1 Biostatistics Department, Harvard School of Public Health, Boston, MA 02115 USA; 2 Channing Division of Network Medicine, Brigham and Women's Hospital and Harvard Medical School, Boston, MA 02115, USA; 3 Division of Pulmonary and Critical Care Medicine, Department of Medicine, Brigham and Women's Hospital and Harvard Medical School, Boston, MA 02115, USA; 4 Division of Epidemiology and Biostatistics, College of Public Health, University of Arizona, Tucson, AZ 85724, USA

## Abstract

The revolution in next-generation sequencing has made obtaining both common and rare high-quality sequence variants across the entire genome feasible. Because researchers are now faced with the analytical challenges of handling a massive amount of genetic variant information from sequencing studies, numerous methods have been developed to assess the impact of both common and rare variants on disease traits. In this report, whole genome sequencing data from Genetic Analysis Workshop 18 was used to compare the power of several methods, considering both family-based and population-based designs, to detect association with variants in the *MAP4 *gene region and on chromosome 3 with blood pressure. To prioritize variants across the genome for testing, variants were first functionally assessed using prediction algorithms and expression quantitative trait loci (eQTLs) data. Four set-based tests in the family-based association tests (FBAT) framework--FBAT-v, FBAT-lmm, FBAT-m, and FBAT-l--were used to analyze 20 pedigrees, and 2 variance component tests, sequence kernel association test (SKAT) and genome-wide complex trait analysis (GCTA), were used with 142 unrelated individuals in the sample. Both set-based and variance-component-based tests had high power and an adequate type I error rate. Of the various FBATs, FBAT-l demonstrated superior performance, indicating the potential for it to be used in rare-variant analysis. The updated FBAT package is available at: http://www.hsph.harvard.edu/fbat/.

## Background

Both existing and novel methods incorporating family-based and population-based designs were compared in this report. All the methods we compare use a single test for a set of multiple single-nucleotide polymorphisms (SNPs) in a region (gene in our setting). This approach avoids the problem of needing large samples for testing rare variants individually.

The term *family-based association tests *(FBAT) refers to a suite of family-based association testing methods that rely on an extension of the transmission disequilibrium test. We used 2 newly developed rare-variant association tests in the framework of FBAT, FBAT-v, FBAT-lmm, and 2 previously existing multimarker FBAT tests, FBAT-m and FBAT-l. Although the Genetic Analysis Workshop 18 (GAW18) sample size is large, it is made up of a small number of pedigrees with a large number of individuals per pedigree. The FBAT approach treats all nuclear families in a pedigree as independent, unless a trait locus is known to be linked to the markers under test. The Q1 variable, which was not simulated to be directly associated with any causal gene, was very highly heritable (60%; Table 1), and failure to adjust using an empirical variance led to inflated type I errors for Q1.

**Table 1:** Heritability and coheritability

	Exam 1	Exam 2	Exam 3
**SBP**	28.6%	22.2%	31.1%
**DBP**	29.4%	30.4%	35.9%
**SBP_DBP**	52.9%	32.0%	73.6%
**Q1**	62.8%	-	-

DBP, diastolic blood pressure; SBP, systolic blood pressure.

We chose to first test the methods on *MAP4*, a gene that was simulated to be associated with blood pressure in the GAW18 data. Then, the most powerful tests that maintained adequate type I error were used on a whole chromosome scan of chromosome 3. Because many of the tests we considered are unable to provide results when using all SNPs, our analysis strategy starts with reducing the number of SNPs based on functional assessment.

## Methods

Variants were filtered based on their predicted function. For coding variants, SnpEff (http://snpEff.sourceforge.net) was used to predict nonsynonymous, splice, and stop variants. Nonsynonymous variants were further classified using polyphen2 [1]. Lymphoblastoid cell line (LCL) expression quantitative trait loci (eQTLs) from Caucasian (CEU) International Haplotype Map Project (HapMap) samples were used to highlight SNPs affecting the transcription of *MAP4 *[2]. Polyphen scores above 0.5 were included together with splice and stop variants in our analysis. An arbitrary cutoff of 3.4 (-log_10 _*p *value from eQTL analysis) was used for eQTL filtering.

FBAT-v [3] and FBAT-lmm (JJ Zhou, MN Laird, personal communications, 2013) are 2 newly developed gene-based rare-variant tests. FBAT-v is analogous to gene-based burden tests developed for case-control studies. FBAT-lmm is a variance component test. Although FBAT-lmm is also a transmission disequilibrium-based test, the trait is modeled through a linear mixed model (LMM), where a random genetic component is introduced and tested. It allows genetic effects within the region to be both protective and deleterious. *P *values are determined using 1000 permutations. FBAT-m [4] and FBAT-l [5] are part of the preexisting FBAT suite of tests that were designed for common variants, but can be used with multiple SNPs. FBAT-m is a multivariate test with degrees of freedom equal to the number of linearly independent SNPs. The linear combination test (FBAT-l) used the noninformative families to estimate the optimal weights for the linear combination of SNPs.

The sequence kernel association test (SKAT) has been proposed as a test for association between both common and rare genetic variants in a region using either continuous or dichotomous traits [6,7] for population designs. Under the semiparametric regression model, a local relationship (similarity), or "kernel" matrix, is estimated using the genotypes from a testing region, for example, identical by state (IBS) kernel and gaussian kernel for nonlinear effects. As described by Yang et al, genome-wide complex trait analysis (GCTA) is a toolkit designed to estimate heritability using genome-wide association studies (GWAS) data from unrelated individuals based on an LMM under a polygenic assumption [8,9]. We have adapted the GCTA approach to test only the SNPs in a gene or region, and, as such, it is comparable to the SKAT approach; indeed, LMM and semiparametric regression share many theoretical connections [10].

## Results

We used the complete set of 200 replicates for assessing type I error and power, using an alpha of 0.05 to determine statistical significance. In our analyses, we focused on 2 continuous phenotypes: systolic blood pressure (SBP) and diastolic blood pressure (DBP). Heritability estimates for SBP and DBP were both in the range of 20% to 30% (see Table 1). Coheritabilities for the 2 traits (i.e., the proportion of phenotypic covariance explained by common genetic covariance) ranged from 30% to 70% for 3 exams (see Table 1). The analyses were adjusted by age, sex, age*sex, and BPmeds (i.e., current use of antihypertensive medications) at each exam by generating standardized residuals. We also analyzed average residuals over 3 exams. For the Q1 phenotype, we adjusted for age and sex only.

### Functional assessment for screening

The *MAP4 *gene encompassed a total of 894 SNPs (Table 2). Of the 894 variants in the *MAP4 *gene, we identified a total of 28 SNPs that met the functional criteria (Tables 2 and 3). Of these, 8 were true causal variants. More than half (57%) of the 28 SNPs were rare (minor allele frequency [MAF] <5%). The same set of functional variants were used for the comparison of both family-based and population-based designs.

**Table 2:** Summary statistics of *MAP4 *gene

# of SNPs	Total	MAF <1%	MAF <5%
**No filtering**	894	613 (68.6%)	742 (83.0%)
**After filtering**	28	9 (32.1%)	16 (57.1%)

**Table 3:** Names and MAF of 28 SNPs that remain for all analyses

SNP	MAF	SNP	MAF	SNP	MAF	SNP	MAF
3-47894286	0.0085	3-47951670	0.0043	3-47956424	0.3590	3-48138082	0.3162
3-47913455	0.0085	3-47952843	0.0214	3-47957741	0	3-48140634	0.3205
3-47933630	0.0128	3-47953352	0.0043	3-47957996	0.0214	3-48413179	0.4017
3-47933903	0.0128	3-47953405	0.3718	3-47958037	0.3120	3-48508585	0.2521
3-47950674	0.0043	3-47953733	0.3162	3-48040283	0.0256	3-48519821	0.2564
3-47950908	0	3-47953813	0.0043	3-48040284	0.0214	3-48520289	0.2222
3-47951458	0	3-47953876	0.0043	3-48123540	0.3162	3-48531227	0.1795

### Family-based analysis

Because of the large number of markers analyzed in a region, FBAT-m did not perform well and the results are not reported. Likewise, results from FBAT-lmm were also omitted, as it currently cannot adjust for multiple families within a pedigree. For the extended pedigree analysis of the *MAP4 *gene region, the empirical variance estimator [3,5,6] is needed to maintain type I error when phenotypes of relatives are highly correlated. Both FBAT-v -e (empirical variance estimator) and FBAT-l highlighted the association of the *MAP4 *gene across all simulation replicates (Table 4). The highest power and the strongest association signal was identified using FBAT-l.

**Table 4:** Type I error and power comparison based on family studies (n = 849).

FBAT -v -e				
	**Exam 1**	**Exam 2**	**Exam 3**	**Average**
	
**SBP**	0.595	0.520	0.420	0.615
**DBP**	0.495	0.395	0.885	0.520
Q1*	0.065			

**FBAT -l**				

	**Exam 1**	**Exam 2**	**Exam 3**	**Average**
	
**SBP**	0.990	0.960	0.910	1
**DBP**	0.980	0.930	0.885	0.995
Q1*	0.03			

The current beta version of FBAT package does not allow -v - e. The results shown in Table 4 (-v -e) were analyzed by an R script that excludes families with parental missing genotypes.

*Q1 is the phenotype simulated for evaluating type I error.

### Population-based analysis

Using 142 unrelated individuals, type I error and power between SKAT and GCTA were compared for association with *MAP4 *(Table 5). Both SKAT-o and SKAT default parameter settings were used. In our analysis, SKAT using the default weighting schemes (weighted by beta[0,25]) has the highest power, which we reported here. Both methods maintain correct type I error. SKAT had slightly higher power in this study, although GCTA had power greater than 85% for all phenotypes tested.

**Table 5:** Type I error and power comparison based on population study (n = 142)

SKAT				
	**Exam 1**	**Exam 2**	**Exam 3**	**Average**
	
**SBP**	0.985	0.98	0.975	0.98
**DBP**	0.92	0.845	0.8	0.97
Q1*	0.06	-	-	-

**GCTA**				

	**Exam 1**	**Exam 2**	**Exam 3**	**Average**
	
**SBP**	0.85	0.645	0.545	0.895
**DBP**	0.69	0.52	0.42	0.825
Q1*	0.055	-	-	-

*Q1 is the phenotype simulated for evaluating type I error.

### Chromosome 3 scan

**Figure 1:**
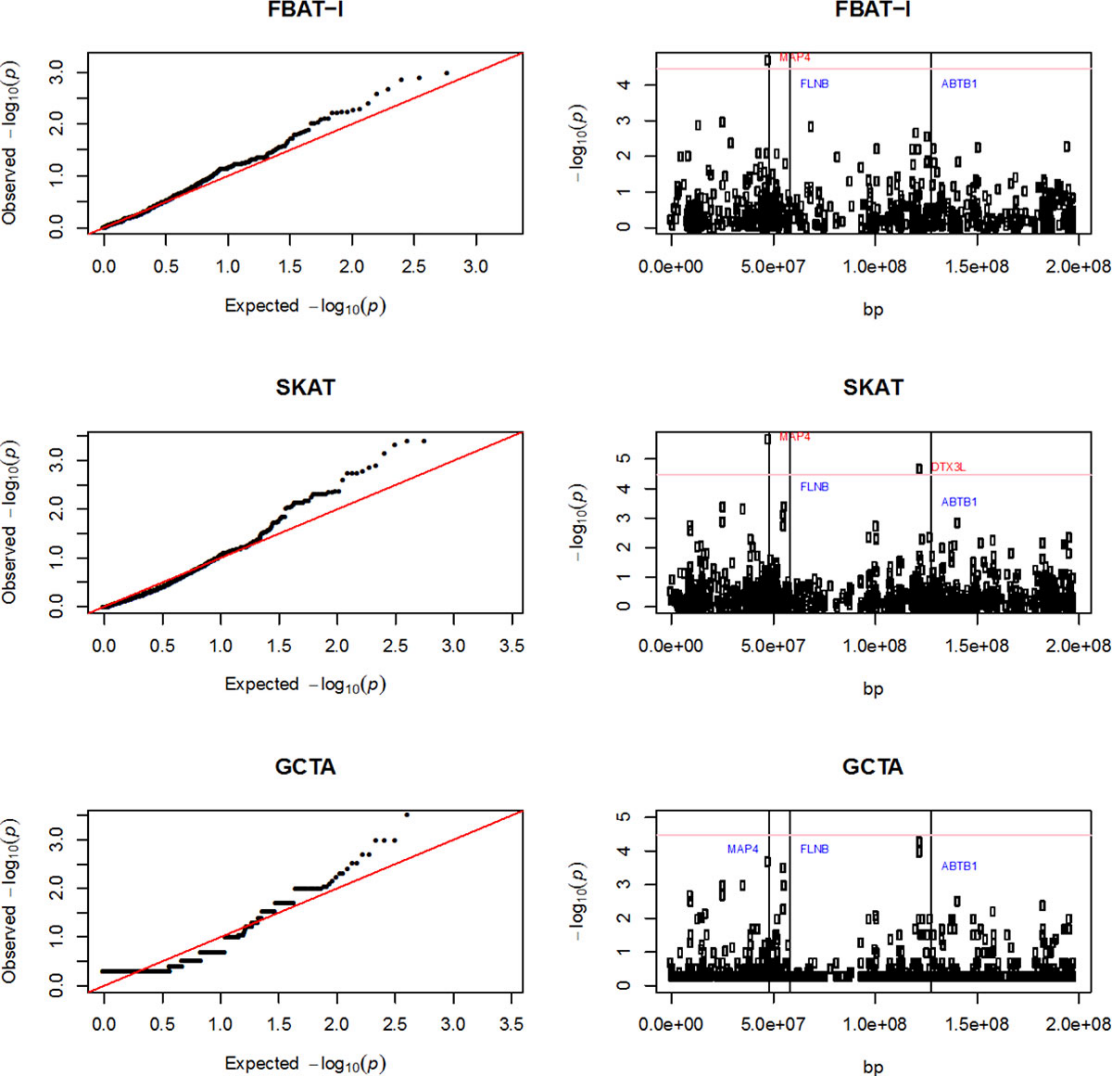
**Genome scan using methods FBAT-l, SKAT, and GCTA**. Average residual of blood pressure over 3 time points was used for association analysis. Vertical line represents causal gene; genes that passed Bonferroni correction threshold are marked by red, the others are in blue.

A whole genome scan was performed using FBAT-l for both family and population-based methods after adjusting for first 10 principal components generated by EIGENSTRAT [11]. Only chromosome 3 was scanned for this manuscript, which is suggested by the GAW18 data description. Genes were defined by transcription start and end positions obtained from the University of California Santa Cruz (UCSC) Genome Browser hg19 build (http://genome.ucsc.edu/). In total, 1443 genes were analyzed for their association with average residual of blood pressure over three time points (Figure 1). The same filtering algorithm used in the analysis of candidate gene *MAP4 *was adopted. Using FBAT-l and SKAT, we identified the *MAP4 *gene as passing the genome-wide significance level. SKAT also identified gene *DTX3L*, which is adjacent to the causal gene *ABTB1*. Although no genes pass genome-wide significant level using GCTA, genes that are the most significant (*MAP4 *and *DTX3L*) overlap with the results from SKAT.

## Discussion

Both family-based and population-based analyses of whole genome sequencing data were evaluated for their power to detect associations with a simulated phenotype with variants in the *MAP4 *gene and on chromosome 3. This approach incorporated the use of functional prediction information to filter variants as would traditionally be done in most applied studies. Both SKAT and GCTA had high power and an adequate type I error rate. Of the various FBAT tests, FBAT-l demonstrated superior performance, indicating the potential to be used in rare-variant analysis. The lack of population substructure and availability of potential phenotypes contribute to the high performance of FBAT-l. Absent these conditions, the performance degrades. The relatively poor performance of FBAT-lmm could be a result of small sample size and concordant direction of effect size across SNPs. However, FBAT-lmm shows promise for the case where effect sizes within a test region vary in signs of risk. It does not currently have the capability to analyze extended pedigrees.

We also note that when analyzing extended pedigree data and highly correlated traits between relatives, the empirical variance estimator (-e) should be used to achieve the correct type I error. However, its use decreases the effective sample size so that it is closer to the number of independent pedigrees. Finally, our analysis demonstrates that using the average phenotype over 3 time points gives higher power compared to single-time-point phenotype analysis. This suggests the combination of the phenotypes from different time points, or even the combination of SBP and DBP, may achieve higher power.

## Conclusions

In this paper, we compared various FBAT region based tests and compared family based tests with population based tests. Our results show that FBAT -l outperformed FBAT -v0 when testing *MAP4 *and this could be due to some causal variants of MAP4 within the variants for analysis being common. Our population-based tests comparison suggests that in the absence of population substructure, the population-based association tests are more powerful.

## Competing interests

The authors declare that they have no competing interests.

## Authors' contributions

JJZ developed FBAT-lmm, ran analyses, and wrote the paper. WKY codeveloped FBAT-v and FBAT-v -e, and assisted in computing. MHC developed the functional screening algorithm for SNP reduction. DQ assisted in data cleaning and formatting, and running of principal components. MLNM edited and revised the manuscript; NML performed the overall design and edited the manuscript. All authors read and approved the final manuscript.
